# The COVID-19 Pandemic Crisis and Patient Safety Culture: A Mixed-Method Study

**DOI:** 10.3390/ijerph19042237

**Published:** 2022-02-16

**Authors:** Ognjen Brborović, Hana Brborović, Leonarda Hrain

**Affiliations:** 1 Department of Social Medicine and Organization of Health Care, Andrija Štampar School of Public Health, School of Medicine, University of Zagreb, 10000 Zagreb, Croatia; obrborov@snz.hr; 2 Department of Environmental and Occupational Health and Sports Medicine, Andrija Štampar School of Public Health, School of Medicine, University of Zagreb, 10000 Zagreb, Croatia; 3 School of Medicine, University of Zagreb, 10000 Zagreb, Croatia; leonarda.hrain7@gmail.com

**Keywords:** patient safety culture, healthcare workers, COVID-19 pandemic, hospital, healthcare safety, healthcare professional safety

## Abstract

The COVID-19 pandemic has put inordinate pressure on frontline healthcare workers (HCWs) and hospitals. HCWs are under chronic emotional stress, affected by burnout, moral distress and interpersonal issues with peers or supervisors during the pandemic. All of these can lead to lower levels of patient safety. The goal of this study was to examine patient safety culture values in a COVID-19 frontline hospital. Patient safety represents action, while patient safety culture represents the beliefs, values and norms of an organization that support and promote patient safety. Patient safety culture is a prerequisite for patient safety. A cross-sectional study on healthcare workers (228, response rate of 81.43%) at a COVID-19 frontline hospital was conducted using the Hospital Survey on Patient Safety Culture (HOSPSC), which had PSC dimensions, single question dimensions and comments. Our research revealed that, during the COVID-19 pandemic, a number of patient safety issues have been identified: low communication openness and current punitive response to errors, which might have incapacitated HCWs in the reporting of adverse events. Although participants expressed high supervisor/management expectations, actual support from the supervisor/management tier was low. Poor teamwork across units was identified as another issue, as well as low staffing. The infrastructure was identified as a potential new PSC dimension. There was a lack of support from supervisors/managers, while HCWs need their supervisors to be available; to be visible on the front line and to create an environment of trust, psychological safety and empowerment.

## 1. Introduction

Healthcare workers (HCWs) are persons serving in healthcare or in social care settings. HCWs are from various groups of professions, like doctors, nurses, technicians, therapists and pharmacists. These include people who are in a training process (medical/nursing/dental students on a clinical placement and other trainees) or whose professional work is in a healthcare setting. They may work in direct contact with patients or not; they may also have the potential of direct or indirect exposure to patients or infectious materials [1,2]. They work in settings where healthcare is delivered. These settings differ in many aspects, for example, whether they are publicly or privately governed and regarding the type of healthcare setting—emergency medicine facilities, hospitals, nursing homes, outpatient clinics, medical offices and so on [1].

Healthcare is becoming more effective and more complex each year [3]. Practice evolves and varies between individuals, as well as between different facilities. As new technologies and treatment options arise, procedures often change without the establishment of standard procedures. This presents new options, as well as additional obligations, for HCWs to adhere to the new conditions. It is expected that all healthcare settings across the world should be effective, efficient, safe, timely, equitable, integrated and people-centered [3]. Working in healthcare is complex, accountable, risky and difficult. Risks at work can affect HCWs’ health. The National Institute for Occupational Safety and Health reported that HCWs face a wide range of risks, including sharp injuries, chemical and drug exposure, back injuries, various occupational allergens, violence and stress. HCWs have the highest rates of nonfatal injuries and occupational diseases of any industry sector [4]. These injuries and diseases affect mental and physical health, resulting in medical issues that can affect health and the ability to work, like occupational contact dermatitis, occupational asthma, occupational cancer, depression, insomnia, burnout and more [4,5]. Some of these occupational diseases and injuries can be fatal. However, most importantly, these occupational risks and consequences are preventable [4].

Although HCWs work is hard, as they strive to provide the same thing—patient treatment and care while maintaining patient safety [1]. Patient safety is the prevention and reduction of risks, errors and harm, also known as adverse effects, which might occur to patients during the provision of healthcare or in healthcare settings [3,4]. Examples include preventing falls in a healthcare facility, as well as the administering of the wrong type or wrong dosage of a medication. Patient safety culture is a prerequisite for a safe patient safety [6]. A culture that emphasizes patient safety is key to ensuring successful outcomes for all patients and their well-being. Patient safety represents action, while patient safety culture includes individual and group values, perceptions, competencies, behavioral patterns and attitudes towards health and safety management in a healthcare organization—in other words, “the way we do things around here” [6,7,8]. These beliefs extend to all levels and every department of a healthcare organization and influence the actions and behaviors of staff throughout it [9]. When PSC is positive, HCW actions towards sustaining PS should be positive and successful [6]. For example, the prevention of falls in a healthcare institution is not merely the provision of non-slippery floors. It has to do to with knowledge of how falls occur, which patients are at risk and how to handle patients in a safe way, as well as the attitudes of HCWs and managers towards prevention.

The COVID-19 pandemic has put excessive pressure on frontline HCWs and hospitals. The higher-than-usual workload, huge number of patients, novel experiences of the pandemic, lack of knowledge about the etiopathogenesis, unpredictability and lethality of the virus, uncertainty about outcomes, shortage of PPE and unavailability of vaccines in many countries and lack of tried-and-tested treatments: all of these and many more conditions have led HCWs to a new, unprecedented situation that has amplified their emotional distress and burnout [10,11,12,13,14]. Uncertainties regarding ICU bed capacity, staff shortages, non-ICU HCWs working in ICU or makeshift ICU, no standard protocols for specific situations or rapidly changing recommendations can all create moral distress in HCWs [10,15,16]. In Croatia, where this research took place, the government introduced measures and recommendations regarding protection measures against the COVID-19 virus [17]. The measures are applied in all healthcare facilities and refer to staff safety issues such as the prevention of virus transmission and personal protective equipment. Healthcare facilities have also adopted guidelines for the treatment of COVID-19 patients at different hospital levels [18,19]. The guidelines might differ between healthcare facilities. However, neither crisis guidelines nor training regarding patient safety in this crisis have been developed. This might further burden HCWs, because new concerns regarding professional liability due to pandemic-related issues might arise [14]. Besides emotional and moral distress, HCWs may experience interpersonal issues with other burdened colleagues, resulting in a lack of the psychological safety that is essential for them to do their job as best they can and maintain patient safety (PS) levels [10]. Another unique aspect of this pandemic—social distancing—might limit the wherewithal and opportunities for HCWs to take care of themselves. Usually, human beings rely on unwinding from work through rest at home, where all the damage caused by work can be repaired. However, this physiological need cannot be met during this pandemic, because human social interactions are considered a risk. Due to the lockdown, people cannot enjoy the activities they usually carry out for relaxation [10]. Moreover, HCWs feel constrained not to spread the virus to their families and friends; therefore, many of them have decided to isolate themselves at home [10,20].

These unique work and life conditions can worsen the existing health conditions and amplify chronic stress, burnout and their consequent effects on HCWs’ work. Their performance, in turn, affects the healthcare quality, possibly resulting in more patient safety adverse events [10,13,15,16,20,21,22,23,24,25]. During these times, HCWs are aware that patient safety must remain sound, which puts immense pressure on them [10].

There is an existing research gap in literature concerning the extent to which the COVID-19 pandemic has affected PSC. The aim of this study was to examine patient safety culture values in a COVID-19 frontline hospital. Our research questions were: what are the values of PSC in a frontline hospital? Which PSC dimensions are affected? What are HCWs’ thoughts on that? PSC dimensions, single question dimensions and comments were comprehensively analyzed.

## 2. Methods

### 2.1. Survey Instrument

The Hospital Survey on PSC questionnaire (HSOPSC) was designed to measure 12 dimensions of PSC (Table 1) [6,7]. The HSOPSC questionnaire contains 42 questions that mainly use the 5-point Likert response scale of agreement (‘Strongly disagree’ to ‘Strongly agree’) or frequency (‘Never’ to ‘Always’). Three of the 12 PSC dimensions (*Feedback and Communication About Error, Communication Openness and Frequency of Events Reported*) use the frequency response option. In comparison, the other nine dimensions use the agreement response option [6,7]. The dimension results can be interpreted either as a percentage or a value, where a number lower than 3 equals weakness, three equals a neutral state and above 3 equals strength. The Safety Grade section requires each participant to assign a grade (a single-item measure scored from 4 (A) to 0 (E)), the higher score indicating a higher patient safety level. Healthcare adverse events reporting is assessed on a single-item scale that asks, “In the past 12 months, how many adverse event reports have you filled out and submitted?”. The response categories range from no adverse event reports (0), 1 to 2 (1), 3 to 5 (2), 6 to 10 (3), 11 to 20 (4), and ≥21 reports (5). The questionnaire has a comments section, which invites the participants to state any thoughts on the matter they have and wish to express [7].

The Croatian translation of the original American HSOPSC showed that 11 dimensions were identified by exploratory factor analysis with acceptable reliability scores compared to the original 12 in the US sample, and five of the 12 dimensions had a Cronbach’s α higher than 0.7, suggesting a reasonable fit to the original HSOPSC. The confirmatory factor analysis confirmed a good fit to the original American model [6,7].

### 2.2. Study Procedure

This study employed the Croatian version of HSOPSC, which was translated and validated and used in our previous research [6,26]. A cross-sectional study was employed, which included doctors and nurses in a COVID-19 pandemic frontline hospital. The hospital was selected using a convenience sample, which took into account the employees’ willingness and consent to take part. The research was conducted at the end of the first year of the COVID-19 pandemic, during the third wave.

Both doctors and nurses were approached. The research was conducted in March and April 2021 and was anonymous and voluntary. The questionnaires were provided in unmarked envelopes along with a consent form. The researchers used morning staff meetings and weekly educational meetings to distribute the questionnaires personally to physicians and nurses who were willing to participate. Upon completion, questionnaires and consent forms were returned in separate sealed and unmarked envelopes. Each respondent then placed the envelopes in a box placed in each department’s nurses’ room. The department head nurses collected the boxes and then returned them to the principal investigator.

### 2.3. Ethical Permission

An approval from the University of Zagreb, School of Medicine Ethical Board was requested, and the study was approved. Approvals from the hospital board of ethics and manager were also solicited and obtained. Participants’ consent was requested and received.

### 2.4. Statistical Analysis

All questionnaires were collected and entered into an electronic database, and the completeness of the data was checked. Since HSOPSC items were worded in both the positive and negative directions, negatively worded items were first reverse-coded (Table 1). The composite scores for PSC were then calculated.

A qualitative analysis of the comments in the questionnaire was performed to identify the participants’ comments. Thematic studies were employed to inductively identify participant issues not addressed in the questionnaire. The comments were analyzed through familiarization, coding, generating themes, reviewing themes and defining and naming themes [27].

Regarding the quantitative analysis, all continuous data were tested for normality using the Shapiro–Wilks test, and significant departures from normality were found for all analyzed variables. For all analyses, statistical significance was set at the *p*-value of <0.05. Chi-square and Fisher’s exact tests were used for categorical variables. The analysis was performed using IBM SPSS Statistics for Windows, version 25.0 (IBM Corp., Armonk, NY, USA).

## 3. Results

A total of 280 questionnaires were distributed to doctors and nurses present at work when the research was conducted. A total of 228 were returned, constituting a fair response rate of 81.43%, and comprising 43 physicians and 185 nurses. The participating physicians and nurses worked in 11 departments: many different hospital units/no specific unit (10.1%), COVID 1 (4.8%), COVID 2 (8.8%), COVID 3 (11.8%), COVID 4 (6.1%), COVID 5 (6.2%), Non-COVID ICU (9.2%), Mixed COVID/Non-COVID Pediatric Department (18.9%), COVID 6 (7%), ER—COVID (12.3%) and the Department for Clinical Microbiology LAB (4.8%). During the COVID-19 pandemic, the already organized and functioning departments (for example, departments of gastroenterology) were reorganized to fully care for COVID patients, becoming COVID departments.

### 3.1. Qualitative Analysis of the Comments Sections

We began our analysis by exploring the comments section. A total of 22 (9.65%), the participants left a comment. After familiarization and coding came the generation of themes. These themes were reviewed, defined and named. Three major themes emerged from the qualitative data, resulting in the identification of three major themes: (1) “Comments Directly Related to Patient Safety Culture Dimensions“, (2) “Assertive Comments” and (3) “Infrastructure” (Table 2).

1. In the “Assertive Comments” theme group, comments were expressed as praise for this research and acknowledgment that the patients were safe in this hospital (Table 2):
  “Praise for the chosen subject. We should talk about patient safety more and change attitudes. It is necessary to put emphasis on, and familiarize employees with, patient safety”;
  “The patients are safe in our hands”.
2. The “Infrastructure” theme (Table 2) revealed concern for the infrastructure of the hospital building, its sanitation (water and power outages) and the state of its equipment. Some of the comments are expressed below and in Table 2:
  “I don’t consider the water drinkable—it is often brown, and it is embarrassing to explain to patients that it is potable when I wouldn’t drink it myself!”;
  “It was challenging to complete this survey given that the Hospital is ancient, worn out, in poor repair and further destroyed by the earthquake”;
  “In addition to the items examined, the hospital’s infrastructure needs to be changed to improve staff working conditions and patient safety!”; “Cribs are without fences (the bed is enclosed with room chairs if necessary), oxygen bottles without stands and insufficiently attached to the frame, so they sometimes roll over on the floor in the patient’s room, the valves on the oxygen bottles loosen and hiss as they run out of rubber, in the external staircase through which staff pass and enter, plaster is falling from the walls, brown tap water”.
3. The “Detailed Answers to Patient Safety Culture Dimensions” theme revealed three subgroups that preoccupied the participants: (1) “Under-reporting of events”, (2) “Staffing and management” and (3) “Communication” (Table 2).

The “Under-reporting of events” subgroup (Table 2) expressed concern that adverse events are underreported; instead of a culture of learning from mistakes and extracting a positive message, there is an existing fear of blame and that mistakes will be punished. They also articulated that systemic problem solving is not performed. They disclosed the following:

“This was, and still is, the taboo theme. Instead of learning from mistakes, extracting some positive message, and changing the mode of operation, such things get hushed up, nobody is informed about them, and the staff are repeatedly warned they will be placed on their superiors’ ‘black list’”;

“Systematic problem solving is not done, only solving individual cases”;

“In my opinion, adverse events are under-reported. If we want things to change, we must know what went wrong!” The “Staffing and Management” subgroup (Table 2) revealed that there is low staffing and that respondents have concerns regarding staff safety and staffing, as well as patient safety in regard to staffing and/or number of patients. They disclosed the following (Table 2):

“Too few nurses, porters and auxiliary staff. We are entirely out of protection. Other institutions have security guards”;

“If he had employed more nurses, patient safety would be higher, and therefore unwanted events fewer”;

“A reduction in the number of patient beds in the department or an increase in the number of nurses in the shift, depending on the severity of the patients’ medical issues, would reduce the number of errors and unwanted events. The patients would be safer”.

The “Communication” subgroup articulated the communications issues with other HCWs, like epidemiologists, who are essential in the pandemic and communication across departments (Table 2). They disclosed the following:

“Better communication with epidemiologists”;

“Medical findings/hospital records should be delivered by mail to the outpatient patients under a unique code”;

“Inadequate communication across the department”.

**Scheme 1:**
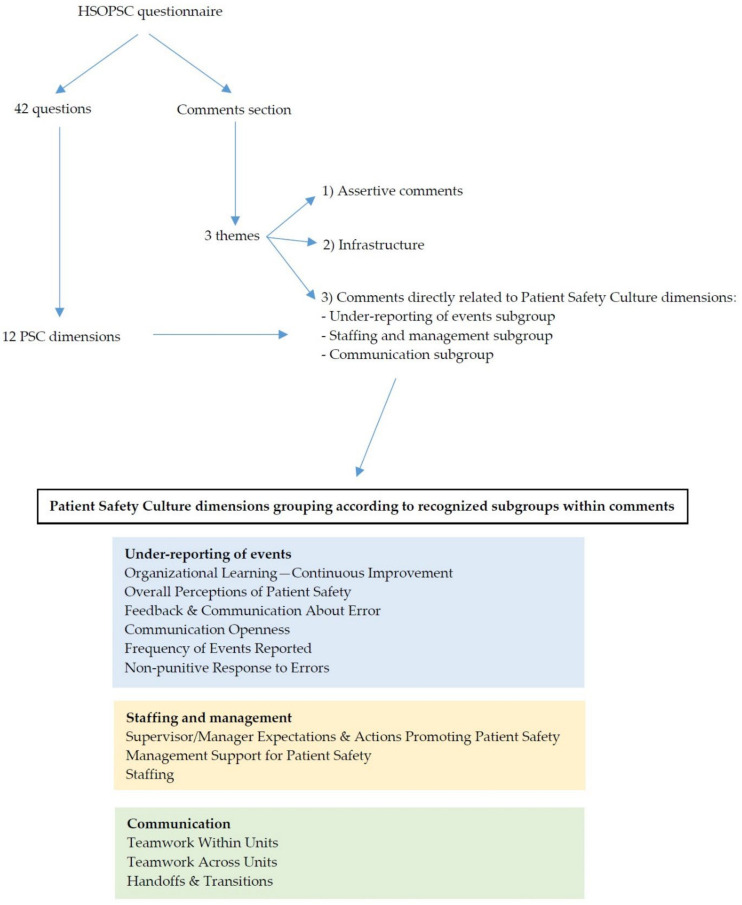
Model of this mixed-method research.

This subgroup of themes and the content comments provided prompted us to do a similar grouping of PSC dimensions and analyze the results (Scheme 1).

### 3.2. Quantitative Analysis—Analysis of the PSC Dimensions Arranged in the Three Subgroups According to the “Detailed Answers to Patient Safety Culture Dimensions” Group from the Qualitative Data Analysis

The PSC dimensions were grouped in the three subgroups identified through the qualitative analysis (Table 3 and Scheme 1). PSC dimension values were analyzed for every department.

**Figure 1:**
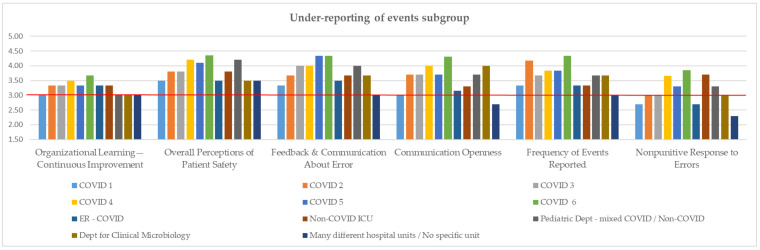
PSC dimension values for the “Under-reporting of events” group across departments.

The “Under-reporting of events” group (Figure 1) revealed that most departments had similar values of PSC dimensions. The lowest values were in three departments: many different hospital units/no specific unit, ER COVID and COVID 1. ER COVID and COVID 1 have been at the front line of the COVID-19 pandemic ever since its beginning. They suffered the longest periods of continuous exposure and were impacted by daily patient admissions visits the most. The respondents who stated they worked at many different hospital units/no specific unit were mainly junior doctors, as well as a few nurses, who had worked at the hospital for periods of 1–5 years. The PSC dimensions with the lowest values in this group were *Communication Openness and Nonpunitive Response to Errors*. The departments with the highest grades were COVID 4 and COVID 6.

**Figure 2:**
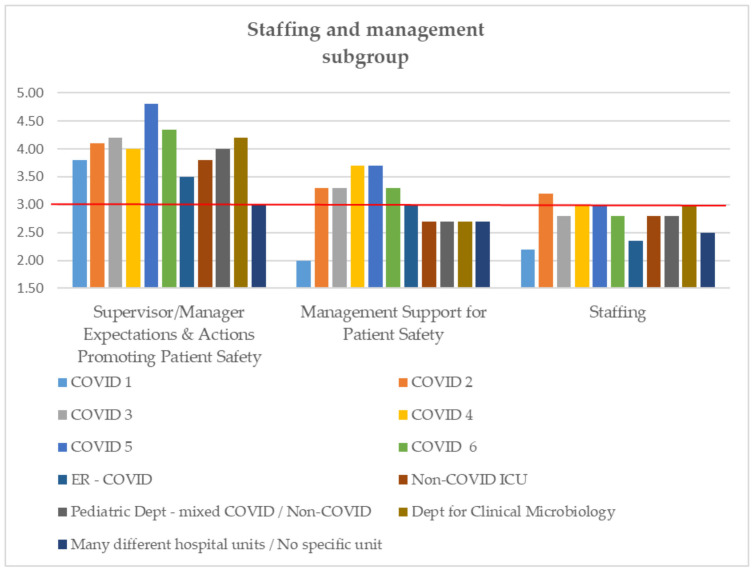
PSC dimension values for the “Staffing and management” group across departments.

The “Staffing and management” group (Figure 2) demonstrated a discrepancy between a high level of *Supervisor/Manager Expectations and Actions Promoting Patient Safety* and low levels of *Management Support for Patient Safety* and *Staffing* at all departments. In addition, the *Staffing* dimension had the lowest levels across all departments.

**Figure 3:**
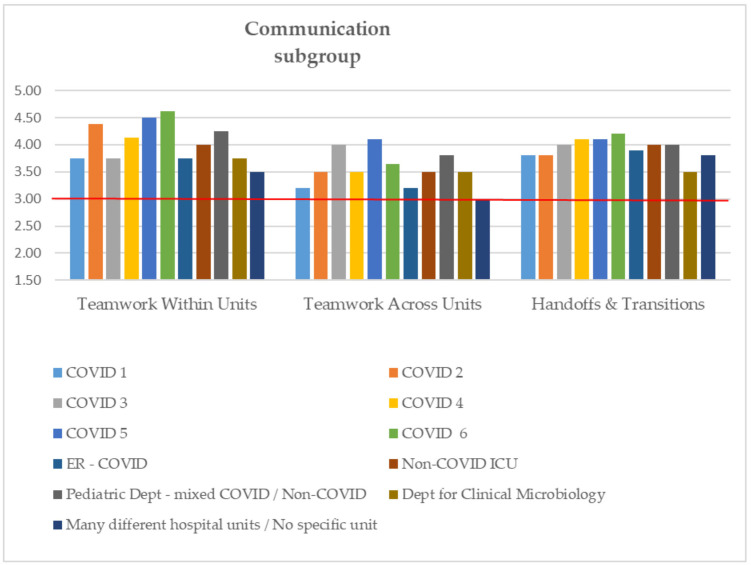
PSC dimension values for the “Communication” group across departments.

The *Communication* group (Figure 3) revealed higher values of *Teamwork Within Units* in almost all departments but low values of *Teamwork Across Units*, while *Handoff and Transition* values were in a middle position.

### 3.3. Single-Question Responses

The Patient Safety grade chosen by the majority of respondents was very good (41.2%) or excellent (28.1%), while 18.9% graded it as acceptable and 11.8% as poor. No statistically significant difference was found.

Most respondents had neither filled out nor submitted any event report in the previous 12 months (62.72%). Of those who had, most of them had filled and reported 1 or 2 reports, while the rest had filled and reported more than three. No statistically significant difference was found.

## 4. Discussion

Our study aimed to assess how patient safety culture was affected by working practices in a frontline hospital from the beginning of the COVID-19 pandemic. Our starting point was the analysis of the comments (rarely analyzed in HSOPSC research), which—in ways we had not anticipated—changed our perspective. The analysis of the comments revealed three groups (“Assertive Comments”, “Infrastructure” and “Detailed Answers to Patient Safety Culture Dimensions”). Further analysis of the detailed answers on patient safety culture dimensions revealed three sub-groups: “Under-reporting of events”, “Staffing and management” and “Communication” (Scheme 1 and Table 2 and Table 3). This subgroup of themes prompted us to do a similar grouping of PSC dimensions and analyze the results. This analysis revealed that the in-depth implications of our research differed from the superficial. On the surface, it might appear that, overall, the PSC levels were good or high. *Patient Safety Grade* (a single-question dimension) was deemed to be very good or excellent by the majority of the respondents. This was congruent with the PSC dimension *Overall Perceptions of Patient Safety*. This dimension had the highest values measured in the majority of the participating departments.

However, this research revealed that the lowest values were in the departments heavily involved in the care of the COVID-19 patients, suggesting that a high workload is associated with lower patient safety culture. The lowest values measured the PSC dimensions *Communication Openness* and *Non-punitive Response to Errors* (“Under-reporting of events” subgroup). This finding was confirmed and in agreement with the single-question PSC dimension *The Number of Events Filed and Reported in the Last 12 Months*—the majority of respondents had neither filed nor reported any event. This was also expressed in the comments of the participants (Table 2). This result supports our previous findings that HCWs do not report events, because they feel guilty and are afraid of legal repercussions if they report their own mistakes [26]. This result is congruent with the Agency for Quality and Accreditation in Health Care, as well as Social Welfare reports from the last few years [28,29]. Patient safety events are rarely reported, but staff safety events are reported [6,28,29]. The issue of under-reporting and the fear of “punishment” and shame were present before and have remained present during the pandemic [30]. Reporting systems must be effective and available in a safe and trusting environment [31]. Event reporting is a critical part of patient safety, which the hospital system should work on. The other part is developing communication structures to enable the organization to learn from its errors and developing problem-solving strategies and communication between individual HCWs [10]. Event reporting and a safe environment in which it can take place is important in routine work, as well in crises like the COVID-19 pandemic. We expect future research to identify whether new patient safety issues have been identified and raised during this pandemic.

The “Staffing and management” subgroup demonstrated low staffing across all departments and the discrepancy between a high level of *Supervisor/Management Expectations* and low levels of management support for these expectations. The Staffing dimension was low in all departments, which was strongly expressed in the comments (Table 2). Our previous research also demonstrated low staffing before the pandemic [6,26,32,33]. Our country, like the rest of the world, faces a HCW shortage, especially in the nursing workforce [10]. The World Health Organization has estimated that, by the year 2030, there will be a shortfall of HCWs, mostly in low- and lower-middle-income countries [34]. Additionally, countries at all levels of socioeconomic development face, to varying degrees, difficulties in the education, employment, deployment, retention and performance of their workforce [34].

All departments revealed the same: HCWs felt that their expectations were high, but the supervisor/management tier provided no support for these expectations. This was also expressed in the comments of the participants (Table 2). Our previous research, before the pandemic, showed low management support in other Croatian hospitals [26,32,33]. These results are troubling, because the pandemic has exacerbated this issue [10]. HCWs experience a lack of support from supervisors/managers. They need their supervisors to be available; visible on the front line and they need them to create an environment of trust, psychological safety and empowerment [10]. When this is the case, HCWs have a safe environment in which to communicate PS concerns to supervisors/managers [10]. Besides supervisors/managers, HCWs need to have safe communication with peers. HCWs on the frontline often need to make trade-offs to deal with mismatches between demand and available capacity and manage competing priorities [10]. This is the possible explanation for our results demonstrating that, while the *Teamwork Within Units* was good, *Teamwork Across Units* was not as good. It might be that the communication between HCWs in the same department is better. The understanding, departmental culture and rules and obligations are familiar to its members. Other departments might have different rules, communication and culture in general, burdened by a new set of COVID-19 rules and trust issues. The same was revealed in the comments, communication across units and with epidemiologists (Table 2). Unexpectedly, *Handoffs and Transitions* values were in the middle position.

The infrastructure theme revealed concern for the infrastructure of the building of the hospital, the quality of its water supply and the state of its equipment. We found this infrastructural theme not only to be valuable to this research but also worthy of endorsing as a new PSC dimension. Safe and fully functional infrastructure is a prerequisite for healthcare delivery and maintaining patient and staff safety. This applies to all healthcare facilities, rural or urban, in developed or developing countries, in crisis or just everyday functioning [35]. The participants in this study expressed concern regarding the infrastructure of the hospital building, its sanitation (water and power outages) and the state of its equipment (Table 2) The infrastructure of the healthcare facility, as well as other infrastructures (gas, power supply and so on) and clean water and sanitation, are crucial for the normal functioning of the healthcare facility and safety. A PSC infrastructure dimension might examine the anticipation, preparedness for and response to potential infrastructure malfunction at the hospital and department levels. For example, it could ask whether the management anticipates problems and how problems are solved regarding deteriorating hospital buildings, if the management takes action in educating staff how to act in certain situations and whether HCWs are trained in how to act in potential crises (for example power outages), the management has guidelines regarding certain situations and the staff are familiar with them sufficiently enough that they know to act.

The departments most involved were working at ER COVID, COVID 1 and many different hospital units/no specific unit. ER COVID and COVID 1 have been at the front line of the COVID-19 pandemic ever since its beginning, continuously exposed for the longest time and most impacted by the number of daily patient admissions. Many different hospital units/no specific units were an option answered mainly via junior doctors, who worked at the hospital between 1 and 5 years under tremendous workloads and endured lower PSC levels. Junior doctors, especially those who work there temporarily or are visiting, may have the courage to honestly report the state of affairs. They are not burdened by opinions or management decisions if they will not be working in the hospital in the long run. Junior doctors have been recognized as valuable young professionals who can play an essential role in shaping a positive safety culture that encourages reporting all safety concerns [30]. Education on the patient safety as a culture change is mandatory. A positive and fair workplace culture, reducing hierarchy, enabling meaningful senior leadership and mentoring, as well as empowering juniors to speak up and be heard might improve patient safety [30,36,37,38].

Limitations of the study include potential fears among participants that their answers will be disclosed, and they will face repercussions.

Recommendations for future research include developing questions for a new HSOPSC dimensions that will examine the infrastructure and sanitation. We also recommend research into the development of patient safety strategies and guidelines for crises in general, especially for the present pandemic. Another recommendation would be identifying potential new patient safety domains and events that might have occurred during the pandemic. Anticipating new patient safety issues and adverse events during crises might help in preventing them in future crisis situations.

## 5. Conclusions

Our research revealed that, during the COVID-19 pandemic, a number of patient safety issues have been identified: *Communication Openness* and *Non-punitive Response to Errors* were low in departments heavily burdened with COVID-19 patients. *Teamwork Across Units* was low, while *Teamwork Within Units* remained high and *Handoffs and Transitions* was in the middle position. *Supervisor/Management Expectations* were high, but support from the supervisor/management tier was low. *Staffing* was low. The infrastructure was identified as a potential new PSC dimension. The future of healthcare requires a safe and supportive environment of trust and empowerment for HCWs, along with support from supervisors/managers and the same supervisors/managers being available and visible on the front line.

## Figures and Tables

**Table 1:** PSC dimensions and questions [6,7].

	Safety Culture Dimensions (Unit Level)	Questions	
1	Supervisor/Manager Expectations and Actions Promoting Safety	B1	My supervisor/manager says a good word when he/she sees a job done according to established patient safety procedures
		B2	My supervisor/manager seriously considers staff suggestions for improving patient safety
		B3r	Whenever pressure builds up, my supervisor/manager wants us to work faster, even if it means taking shortcuts (reverse worded)
		B4r	My supervisor/manager overlooks patient safety problems that happen over and over (reverse worded)
2	Organizational Learning—Continuous Improvement	A6	We are actively doing things to improve patient safety
		A9	Mistakes have led to positive changes here
		A13	After we make changes to improve patient safety, we evaluate their effectiveness
3	Teamwork Within Hospital Units	A1	People support one another in this unit
		A3	When a lot of work needs to be done quickly, we work together as a team to get the work done
		A4	In this unit, people treat each other with respect.
		A11	When one area in this unit gets really busy, others help out
4	Communication Openness	C2	Staff will freely speak up if they see something that may negatively affect patient care
		C4	Staff feel free to question the decisions or actions of those with more authority
		C6r	Staff are afraid to ask questions when something does not seem right (reverse worded)
5	Feedback and Communication About Error	C1	We are given feedback about changes put into place based on event reports
		C3	We are informed about errors that happen in this unit
		C5	In this unit, we discuss ways to prevent errors from happening again
6	Non-punitive Response To Error	A8r	Staff feel like their mistakes are held against them (reverse worded)
		A12r	When an event is reported, it feels like the person is being written up, not the problem (reverse worded)
		A16r	Staff worry that mistakes they make are kept in their personnel file (reverse worded)
7	Staffing	A2	We have enough staff to handle the workload.
		A5r	Staff in this unit work longer hours than is best for patient care (reverse worded)
		A7r	We use more agency/temporary staff than is best for patient care (reverse worded)
		A14r	We work in “crisis mode,” trying to do too much, too quickly (reverse worded)
8	Hospital Management Support for Patient Safety	F1	Hospital management provides a work climate that promotes patient safety
		F8	The actions of hospital management show that patient safety is a top priority
		F9r	Hospital management seems interested in patient safety only after an adverse event happens (reverse worded)
	Safety Culture Dimensions (Hospital-wide)		
9	Teamwork Across Hospital Units	F4	There is good cooperation among hospital units that need to work together
		F10	Hospital units work well together to provide the best care for patients
		F2r	Hospital units do not coordinate well with each other (reverse worded)
		F6r	It is often unpleasant to work with staff from other hospital units (reverse worded)
10	Hospital Handoffs & Transitions	F3r	Things “fall between the cracks” when transferring patients from one unit to another (reverse worded)
		F5r	Important patient care information is often lost during shift changes (reverse worded)
		F7r	Problems often occur in the exchange of information across hospital units (reverse worded)
		F11r	Shift changes are problematic for patients in this hospital (reverse worded)
	Outcome Measures		
11	Frequency of Event Reporting	D1	When a mistake is made, but is caught and corrected before affecting the patient, how often is this reported?
		D2	When a mistake is made, but has no potential to harm the patient, how often is this reported?
		D3	When a mistake is made that could harm the patient, but does not, how often is this reported?
12	Overall Perceptions of Safety	A15	Patient safety is never sacrificed to get more work done
		A18	Our procedures and systems are good at preventing errors from happening
		A10r	It is just by chance that more serious mistakes don’t happen around here (reverse worded)
		A17r	We have patient safety problems in this unit (reverse worded)
	Patient Safety Grade *	E1	Please give your work area/unit in this hospital an overall grade on patient safety
	Number of Events Reported **	G1	In the past 12 months, how many event reports have you filled out and submitted?

* Single-item measure—grades A through E as response category. ** Single-item measure—numeric response categories.

**Table 2:** Qualitative analysis of the comments sections.

Themes (Groups)	Subgroups	Comments
Comments Directly Related to Patient Safety Culture Dimensions	Under-reporting of events	“This was, and still is, the taboo theme. Instead of learning from mistakes, extracting some positive message, and changing the mode of operation, such things get hushed up, nobody is informed about them, and the staff are repeatedly warned they will be placed on their superiors’ ‘black list’”; “Systematic problem solving is not done, only solving individual cases.” “In my opinion, adverse events are under-reported. If we want things to change, we must know what went wrong!”
	Staffing and management	“Too few nurses, porters and auxiliary staff. We are entirely out of protection. Other institutions have security guards”; “If he had employed more nurses, patient safety would be higher, and therefore unwanted events fewer”; “A reduction in the number of patient beds in the department or an increase in the number of nurses in the shift, depending on the severity of the patients’ medical issues, would reduce the number of errors and unwanted events. The patients would be safer”
	Communication	“Better communication with epidemiologists”; “Medical findings/hospital records should be delivered by mail to the outpatient patients under a unique code”; “Inadequate communication across the department”
Infrastructure		“I don’t consider the water drinkable—it is often brown, and it is embarrassing to explain to patients that it is potable when I wouldn’t drink it myself!”; “It was challenging to complete this survey given that the Hospital is ancient, worn out, in poor repair and further destroyed by the earthquake.”; “In addition to the items examined, the hospital’s infrastructure needs to be changed to improve staff working conditions and patient safety!”; “Occasionally there is a power outage”; “Very often, security is at stake because of the infrastructure barrier.”; “Cribs are without fences (the bed is enclosed with room chairs if necessary), oxygen bottles without stands and insufficiently attached to the frame, so they sometimes roll over on the floor in the patient’s room, the valves on the oxygen bottles loosen and hiss as they run out of rubber, in the external staircase through which staff pass and enter, plaster is falling from the walls, brown tap water”
Assertive Comments		“Praise for the chosen subject. We should talk about patient safety more and change attitudes. It is necessary to put emphasis on, and familiarize employees with, patient safety”; “The patients are safe in our hands”

**Table 3:** PSC dimensions grouped in the three subgroups identified through the qualitative analysis.

Themes (Groups)	Subgroups	Recognized PSC Dimensions
Comments Directly Related to Patient Safety Culture Dimensions	Under-reporting of events	Organizational Learning—Continuous Improvement
		Overall Perceptions of Patient Safety
		Feedback & Communication About Errors
		Communication Openness
		Frequency of Events Reported
		Non-punitive Response to Errors
	Staffing and management	Supervisor/Manager Expectations & Actions Promoting Patient Safety
		Management Support for Patient Safety
		Staffing
	Communication	Teamwork Within Units
		Teamwork Across Units
		Handoffs & Transitions
Infrastructure		Possible new PSC dimension
Assertive Comments		/

PSC—patient safety culture.

## Data Availability

Data are available upon reasonable request from the authors.

## References

[1] Centers for Disease Control and Prevention Appendix 2. Terminology. https://www.cdc.gov/infectioncontrol/guidelines/healthcare-personnel/appendix/terminology.html.

[2] Health Protection Surveillance Centre Definition of a Healthcare Worker. https://www.hpsc.ie/notifiablediseases/casedefinitions/healthcareworkerdefinition/.

[3] WHO Patient Safety. https://www.euro.who.int/en/health-topics/Health-systems/patient-safety/patient-safety.

[4] Centers for Disease Control and Prevention, The National Institute for Occupational Safety and Health (NIOSH) Healthcare Workers. https://www.cdc.gov/niosh/topics/healthcare/default.html.

[5] HZJZ Hrvatski Zavod za Javno Zdravstvo—Služba za Medicinu Rada. Profesionalne Bolesti u Republici Hrvatskoj. http://www.hzzzsr.hr/index.php/porefesionalne-bolesti-i-ozljede-na-radu/profesionalne-bolesti/profesionalne-bolesti-u-republici-hrvatskoj/.

[6] Brborović H., Šklebar I., Brborović O., Brumen V., Mustajbegović J. (2014). Development of a Croatian version of the US Hospital Survey on Patient Safety Culture questionnaire: Dimensionality and psychometric properties. Postgrad. Med. J..

[7] Nieva V.F., Sorra J. (2003). Safety culture assessment: A tool for improving patient safety in healthcare organizations. Qual. Saf. Health Care.

[8] Pronovost P., Sexton B. (2005). Assessing safety culture: Guidelines and recommendations. Qual. Saf. Health Care.

[9] Culture of Safety: An Overview. https://www.ecri.org/components/HRC/Pages/RiskQual21.aspx#:~:text=Patient%20safety%20culture%20is%20the,of%20staff%20throughout%20the%20organization.

[10] Rangachari P., Woods J.L. (2020). Preserving Organizational Resilience, Patient Safety, and Staff Retention during COVID-19 Requires a Holistic Consideration of the Psychological Safety of Healthcare Workers. Int. J. Environ. Res. Public Health.

[11] The COVID-19 Crisis Too Few Are Talking about: Health Care Workers’ Mental Health. https://www.statnews.com/2020/04/03/the-covid-19-crisis-too-few-are-talking-about-health-careworkers-mental-health/.

[12] Lai J., Ma S., Wang Y., Cai Z., Hu J., Wei N., Wu J., Du H., Chen T., Li R. (2020). Factors associated with mental health outcomes among health care workers exposed to coronavirus disease 2019. JAMA Netw. Open.

[13] Santarone K., McKenney M., Elkbuli A. (2020). Preserving mental health and resilience in frontline healthcare workers during COVID-19. Am. J. Emerg. Med..

[14] Bolcato M., Aurilio M.T., Aprile A., Di Mizio G., Della Pietra B., Feola A. (2021). Take-Home Messages from the COVID-19 Pandemic: Strengths and Pitfalls of the Italian National Health Service from a Medico-Legal Point of View. Healthcare.

[15] In Fight against COVID-19, Nurses Face High-Stakes Decisions, Moral Distress. https://hub.jhu.edu/2020/04/06/covid-nursing-cynda-rushton-qa/.

[16] Clark L., Stephens A.F., Liao S., Byrne T.J., Gregory S.D. (2020). Coping with COVID-19: Ventilator splitting with differential driving pressure using standard hospital equipment. Anesthesia.

[17] Government of the Republic of Croatia Coronavirus Protection Measures. https://vlada.gov.hr/coronavirus-protection-measures/28950.

[18] Koronavirus.hr Smjernice za Liječenje Oboljelih od COVID-19. https://www.koronavirus.hr/smjernice-za-lijecenje-oboljelih-od-covid-19/805.

[19] Ministarstvo Zdravstva Republike Hrvatske Smjernice za Liječenje Oboljelih od Koronavirusne Bolesti 2019 (COVID-19) Verzija 3 od 21. Listopada 2021. https://www.hzjz.hr/wp-content/uploads/2021/11/Smjernice-za-lije%C4%8Denje-oboljelih-od-koronavirusne-bolesti-2019-COVID-19-verzija-3-od-21-listopada-2021.-godine.pdf.

[20] The Great Unknown: How Many Health CareWorkers Have Coronavirus?. https://www.usnews.com/news/national-news/articles/2020-04-03/how-many-health-care-workers-have-coronavirus.

[21] Huang J., Liu F., Teng Z., Chen J., Zhao J., Wang X., Wu R. (2020). Care for the psychological status of frontline medical staff fighting against COVID-19. Clin. Infect. Dis..

[22] Chen Q., Liang M., Li Y., Guo J., Fei D., Wang L., He L., Sheng C., Cai Y., Li X. (2020). Mental health care for medical staff in China during the COVID-19 outbreak. Lancet Psychiatry.

[23] Sani G., Janiri D., Di Nicola M., Janiri L., Ferretti S., Chieffo D. (2020). Mental health during and after the COVID-19 emergency in Italy. Psychiatry Clin. Neurosci..

[24] Jun J., Tucker S., Melnyk B. (2020). Clinician mental health and well-being during global healthcare crisis: Evidence learned from prior epidemics for COVID-19 pandemic. Worldviews Evid. Based Nurs..

[25] Shanafelt T., Ripp J., Trockel M. (2020). Understanding and addressing sources of anxiety among health care professionals during the COVID-19 pandemic. JAMA.

[26] Brborović O., Brborović H., Nola I.A., Milošević M. (2019). Culture of Blame—An Ongoing Burden for Doctors and Patient Safety. Int. J. Environ. Res. Public Health.

[27] Braun V., Clarke V. (2006). Using thematic analysis in psychology. Qual. Res. Psychol..

[28] Mesarić J., Mandelsamen Perica M., Hađić Kostrenčić K. (2018). Izvješće o Neočekivanim Neželjenim Događajima za 2017. Godinu.

[29] Mesarić J., Hadžić-Kostrenčić K., Frketić D. Izviješće o Radu Povjerenstva za Kvalitetu Zdravstvene Ustanove 2017. http://www.aaz.hr/sites/default/files/Izvjesce_o_radu_povjerenstva_2017.pdf.

[30] Denning M., Goh E.T., Scott A., Martin G., Markar S., Flott K., Mason S., Przybylowicz J., Almonte M., Clarke J. (2020). What Has Been the Impact of COVID-19 on Safety Culture? A Case Study from a Large Metropolitan Healthcare Trust. Int. J. Environ. Res. Public Health.

[31] Hooper P., Kocman D., Carr S., Tarrant C. (2015). Junior doctors’ views on reporting concerns about patient safety: A qualitative study. Postgrad. Med. J..

[32] Šklebar I., Mustajbegović J., Šklebar D., Cesarik M., Milošević M., Brborović H., Šporčić K., Petrić P., Husedžinović I. (2016). How to Improve Patient Safety Culture in Croatian Hospitals?. Acta Clin. Croat..

[33] Brborović H., Brborović O. (2017). Patient safety culture shapes presenteeism and absenteeism: A cross-sectional study among Croatian healthcare workers. Arhiv za Higijenu Rada i Toksikologiju.

[34] Health Workforce. https://www.who.int/health-topics/health-workforce#tab=tab_1.

[35] United Nations University, Institute for Environment and Human Security Five Things You Need to Know about Health Infrastructures. https://ehs.unu.edu/news/news/five-things-you-need-to-know-about-health-infrastructures.html.

[36] Ward M., Ní Shé É., De Brún A., Korpos C., Hamza M., Burke E., Duffy A., Egan K., Geary U., Holland C. (2019). The co-design, implementation and evaluation of a serious board game ‘PlayDecide patient safety’ to educate junior doctors about patient safety and the importance of reporting safety concerns. BMC Med. Educ..

[37] McCarthy S.E., O’Boyle C.A., O’Shaughnessy A., Walsh G. (2016). Online patient safety education programme for junior doctors: Is it worthwhile?. Ir. J. Med. Sci..

[38] Brennan P.A., Davidson M. (2019). Improving patient safety: We need to reduce hierarchy and empower junior doctors to speak up. BMJ.

